# A Pan-Cancer Analysis of Clinical Prognosis and Immune Infiltration of CKS1B in Human Tumors

**DOI:** 10.1155/2021/5862941

**Published:** 2021-11-20

**Authors:** Yan Jia, Quan Tian, Kaitai Yang, Yi Liu, Yanfeng Liu

**Affiliations:** ^1^Department of Hematology, Xiangya Hospital, Central South University, Changsha, China; ^2^Department of Reproductive Medicine, The Affiliated Hospital of Qingdao University, Qingdao, China

## Abstract

Although more and more evidence supports CDC28 protein kinase subunit 1B (CKS1B) is involved significantly in the development of human cancers, most of the researches have focused on a single disease, and pan-cancer studies conducted from a holistic perspective of different tumor sources have not been reported yet. Here, for the first time, we investigated the potential oncogenic and prognostic role of CKS1B across 33 tumors based on public databases and further verified it in a small scale by RNA sequencing or quantitative real-time PCR. CKS1B was generally highly expressed in a majority of tumors and had a notable correlation with the prognosis of patients, but its prognostic significance in different tumors was not exactly the same. In addition, CKS1B expression was also closely related to the infiltration of cancer-associated fibroblasts in tumors such as breast invasive carcinoma, kidney chromophobe, lung adenocarcinoma, and tumor-infiltrating lymphocytes in tumors such as glioblastoma multiforme, bladder urothelial carcinoma, and brain lower grade glioma. Moreover, reduced CKS1B methylation was observed in certain tumors, for example, adrenocortical carcinoma. Cell cycle and kinase activity regulation and PI3K-Akt signaling pathway were found to be involved in the functional mechanism of CKS1B. In conclusion, our first pan-cancer analysis of CKS1B contributes to a better overall understanding of CKS1B and may provide a new target for future cancer therapy.

## 1. Introduction

Recently, the International Agency for Research on Cancer (IARC) of the World Health Organization (WHO) released the latest global cancer burden data for 2020, which estimated the incidence, mortality, and development trends of 36 cancer types in 185 countries. Based on this statistic, the number of new cancer cases worldwide in 2020 is estimated to be 19.29 million, of which 10.06 million are males and 9.23 million are females. The global cancer death in 2020 is estimated to be 9.96 million, of which 5.53 million are males and 4.43 million are females. On average, about 12,500 people every day, or about 8.7 people every minute, are diagnosed with cancer [1]. In addition, according to this data, by 2020, China will have 4.57 million new cancers (23.7% of the world) and 3 million cancer deaths (30.1% of the world). Compared with other countries, China's cancer incidence and mortality rank first in the world [2]. Behind these figures is the high cost of treatment. According to a survey conducted by the National Cancer Center of China, the average medical expenditure for each cancer patient is RMB 63,000 yuan, while the average annual household income of those surveyed is only RMB 55,000 yuan. As a result, burden of disease is quite heavy [3, 4].

It is well known that the pathogenesis of cancer is very complex. Despite all the difficulties, scientists never give up fighting it. However, limited by various factors, such as small sample size, low statistical power, and poor repeatability, the application of many research results has encountered obstacles [5]. With the continuous deepening of genomics research, oncomolecularbiology has gradually entered the pan-cancer stage. Pan-cancer research refers to simultaneous analysis of multiple different types of tumor genomes to find common characteristics from different sources, so as to help people better understand tumors and provide broad-spectrum targets for clinical diagnosis and treatment [6]. The Cancer Genome Atlas (TCGA) is a tumor genome project launched in 2006 by the National Cancer Institute and the National Human Genome Institute. It aims to use high-throughput genome sequencing, combined with multidimensional data integration analysis, draw a map of tumor genome variation and gene expression, elucidate the mechanism of tumor occurrence and development, adjust diagnosis/classification criteria on this basis, and outline new cancer prevention strategy. At present, TCGA already contains information such as sequencing results, transcriptome analysis, copy number variation, DNA methylation, and single nucleotide variation, covering 33 tumor types [7]. ONCOMINE is one of the largest oncogene microarray databases and comprehensive data mining platforms, which integrates RNA and DNA sequencing data from GEO, TCGA, and published literature. Up to now, the database contains a total of 715 gene expression datasets and 86,733 human tumor/normal tissue samples and is still being updated [8]. The functional genomics data sets of different tumors contained in various public databases provide convenient tools for pan-cancer research.

CDC28 protein kinase subunit 1B (CKS1B) is an indispensable regulatory unit of SCFSkp2 ubiquitin-linked enzyme complex, which promotes the binding of SCF to cyclin inhibitor P27 Kip1 and eventually degrades P27 Kip1, leading to the cell transition from G1 phase to S phase [9, 10]. Beyond that, CKS1B also participates in the degradation of p57, p21, p130, CDT-1, RAG2, h-ORC, and UBP4, suggesting CKS1B is not only involved in cell cycle regulation but also in other molecular events such as transcription, DNA damage repair, cell proliferation and differentiation, cell senescence and apoptosis, and protein secretion and transportation [11]. In recent years, an increasing number of domestic and foreign scholars have discovered that CKS1B is closely related to tumors. For example, in prostate cancer, gastric cancer, lung cancer, multiple myeloma, and ovarian cancer, it was observed to be significantly upregulated [12–14]. Besides, in colon cancer and breast cancer, CKS1B was found to be negatively correlated with prognosis [15, 16]. However, there is still no evidence of pan-cancer researches.

In this study, TCGA, ONCOMINE, and other databases were used for the first time to conduct a pan-cancer analysis of CKS1B. At the same time, we investigated the potential mechanisms of CKS1B in pathogenesis and clinical prognosis of different cancers in terms of gene expression, gene alteration, patient survival, DNA methylation, immune infiltration, and pathway enrichment.

## 2. Materials and Methods

### 2.1. Gene Expression Analysis

The mRNA expression of CKS1B in different tumor types was analyzed in ONCOMINE database, under the settings of *p* value cutoff = 0.001 and fold change cutoff = 1.5. The protein expression of CKS1B in paired samples was explored in UALCAN portal. CKS1B expression difference between tumor and adjacent normal tissues was analyzed in UCSC XENA platform. Available datasets for six tumors, namely, breast cancer, colon cancer, lung adenocarcinoma, ovarian cancer, clear cell renal cell carcinoma, and uterine corpus endometrial carcinoma, were finally selected. The distribution and cellular localization of CKS1B was observed by immunohistochemistry images using Human Protein Atlas (THPA). The violin plots of CKS1B expression in different pathological stages (stage I-IV) of TCGA tumors were obtained by “Pathological Stage Plot” module of GEPIA2.

### 2.2. Survival Prognosis Analysis

The “Survival Map” and “Survival Analysis” module of GEPIA2 were used to make OS (overall survival) and DFS (disease-free survival) analysis diagrams of CKS1B across all TCGA tumors. The log-rank test was used for hypothesis testing, and the threshold was set as a Cox *p* value less than 0.05. R software (version 3.25.0) with the “forest plot” package was utilized to summarize and visualize the survival analysis from PrognoScan.

### 2.3. Bone Marrow Samples and RNA Sequencing

Total RNA was extracted from bone marrow mononuclear cells of acute myeloid leukemia patients or hematopoietic stem cell transplantation donors using Trizol reagent (Ambion, Inc., Carlsbad, CA, USA). Samples were analyzed and quality controlled by BGI Gene Technology Company (China). After passing this test, cDNA library was constructed according to the TruSeq RNA Sample Preparation Kit (Illumina, San Diego, CA, USA). Each library was sequenced using single-reads on a HiSeq2000/1000 (Illumina). Cufflinks were used to measure gene expression levels in RPKM (reads per kilobase per million mapped reads).

### 2.4. Quantitative Real-Time PCR (RT-qPCR)

Total RNA extraction of brain tissues from GEM patients and quantitative real-time PCR reaction was performed using Fast 200 Kit (Feijie Biotechnology Co., Ltd., Shanghai, China) and One Step TB Green PrimeScript RT-PCR kit (MBI Fermentas, St. Leon-Roth, Germany), respectively. The specific operation steps were carried out in accordance with instructions. Relative expression levels of transcription products were normalized to GAPDH. The primer sequences were used as CKS1B-F: 5′-GGACAAATACGACGACGAGGA-3′ and CKS1B-R: 5′-CTGACTCTGCTGAACGCCAAG-3′ and GAPDH-F: 5′-CACCCTGTTGCTGTAGCCAAA-3′ and GAPDH-R: 5′-CACCCTGTTGCTGTAGCCAAA-3′. Conditions for PCR were 30 cycles of denaturation (94°C, 1 min), annealing (60°C, 45 s), extension (72°C, 30 s), and one cycle of final extension (72°C, 10 min).

### 2.5. Immune Infiltration Analysis

The interactive online databases TIMER and GEPIA2 were used to study the relationship between CKS1B expression and abundance of immune infiltration in tumors. B cells, CD4+ T cells, CD8+ T cells, and cancer-associated fibroblasts (CAFs) were selected as research parameters. XCELL, MCPCOUNTER, TIDE, and EPIC algorithms were applied for immune infiltration estimations. *p* values and partial correlation (cor) values were obtained via the purity-adjusted Spearman's rank correlation test. Data were visualized as heat maps and scatter plots. In addition, Pearson correlation analysis was performed to evaluate the level of tumor-infiltrating lymphocytes (TILs). To further investigate the association between CKS1B and immune cell movement and regulation, we also assessed chemokines/chemokine receptors and immunosuppressive factor/immunoactivating factor profiles based on “Chemokine” and “Immunomodulator” modules of TISIDB web portal.

### 2.6. Gene Enrichment Analysis

The protein name “CKS1B” and organism “Homo sapiens” were entered into STRING website. The specific parameters were set as follows: network type (“full network”), meaning of network edges (“evidence”), active interaction sources (“experiments”), minimum required interaction score (“low confidence (0.40)”), maximum number of interactors to show (“no more than 50 interactors” in 1st shell). As a result, the available CKS1B binding proteins were identified. Subsequently, GeneMANIA was applied to do a protein interaction network. Next, Jvenn was used for cross-analysis to screen out common proteins and represented them as Venn diagram. In combination with KEGG (Kyoto Encyclopedia of Genes and Genomes), GO (Gene Ontology) database, and “ggplot2” R package, the enrichment pathway was obtained and visualized. Moreover, the heat maps of selected genes were provided by “Gene_Corr” module of TIMER2, which included cor and *p* values from the purity adjusted Spearman rank correlation test. Finally, the “Correlation Analysis” module of GEPIA2 was used to perform a pairwise gene Pearson correlation analysis of CKS1B and selected genes, and the log2 TPM was applied for dot plots. GSEA (gene set enrichment analysis) was performed using the clusterProfiler package in R.∣ES | >1, *p* < 0.05, and FDR < 0.25 were considered statistically significant.

### 2.7. Genetic Alteration Analysis

The “TCGA Pan Cancer Atlas Studies” in “Quickselect” section of cBioPortal web was logged, and keyword “CKS1B” was entered to check the gene variation characteristics. The results of change frequency, mutation type, and CNA (copy number change) for all TCGA tumors were observed in “Cancer Type Summary” module. The mutation site information of CKS1B can be displayed in the schematic map of protein structure or 3D structure via the “Mutations” module. Kaplan-Meier plots with log-rank *p* values were generated using the “Comparison” module to obtain data on the overall, disease-free, and progression-free survival differences in tumor cases that with and without CKS1B gene alterations.

### 2.8. Methylation Analysis

The methylation status of CKS1B in tumor and adjacent normal tissues was assessed by DiseaseMeth database (version 2.0). The relationship between CKS1B expression and its DNA methylation was investigated using MEXPRESS database.

## 3. Results

### 3.1. CKS1B Is Highly Expressed in Most Types of Human Cancers and Related to Disease Progression

**Figure 1:**
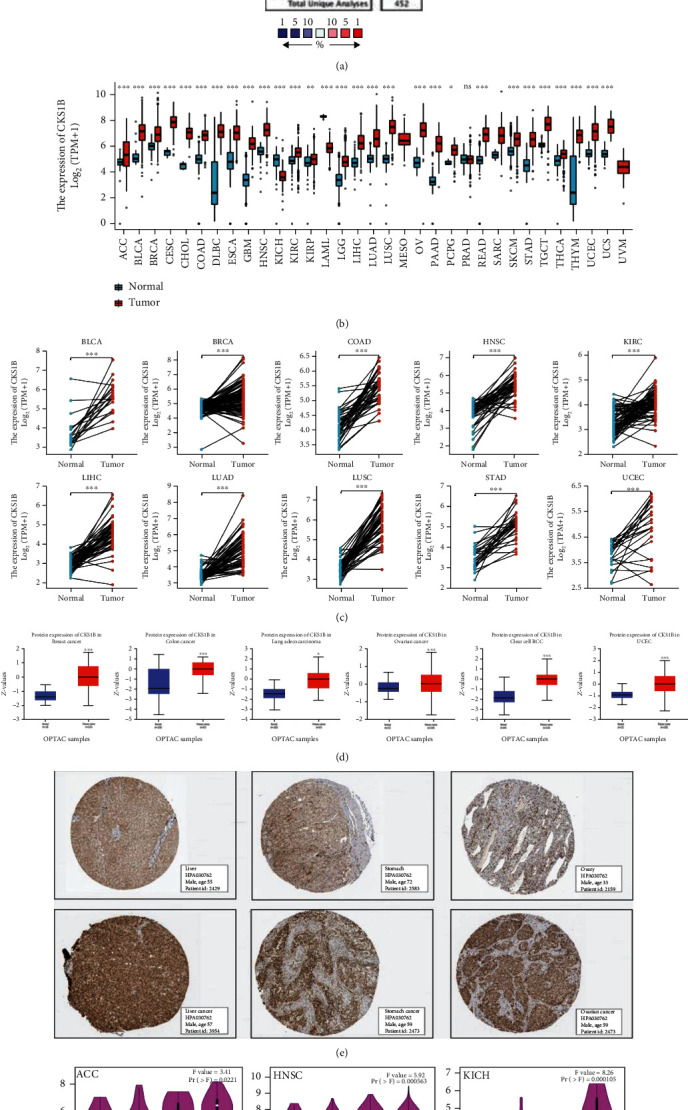
Expression level of CKS1B in different tumors and its relationship with pathological stages. ((a) and (b)) CKS1B expression in different tumors based on ONCOMINE and UCSC XENA. (c) CKS1B mRNA expression in paired tumor tissues and normal tissues based on TCGA. (d) CKS1B protein expression in normal and diseased tissues of breast cancer, colon cancer, lung adenocarcinoma, ovarian cancer, clear cell RCC, and UCEC. (e) Representative immunohistochemistry images and detailed information of CKS1B expression in liver cancer, stomach cancer, ovary cancer tissues, and normal tissues based on THPA. (f) Correlations between CKS1B and tumor stages in ACC, HNSC, KICH, KIPR, LUAD, and PAAD patients based on GEPIA2. ^∗^*p* < 0.05; ^∗∗^*p* < 0.01; ^∗∗∗^*p* < 0.001.

We first analyzed basal expression levels of CKS1B in different blood cells, tumor cell lines, and tumor tissues using Consensus database. As shown in Figure S1A-C, CKS1B was expressed in almost all detected cells and tissues, suggesting it had low cell and tissue type specificity. Then, based on ONCOMINE and UCSC XENA data platform, we found a total of 31 tumors with normal (or highly limited normal) control, of which 29 had statistically differences in the expression level of CKS1B (*p* < 0.05). More specifically, CKS1B was remarkably higher in all 26 tumors than normal tissues, except KICH (kidney chromophobe), LAML (acute myeloid leukemia), and PRAD (prostate adenocarcinoma) (Figures 1(a) and 1(b)). CKS1B expression in paired samples was shown in Figure 1(c) and Figure S1D. Meanwhile, through UALCAN and THPA websites, we found CKS1B protein in BRCA (breast invasive carcinoma), COAD (colon adenocarcinoma), LUAD (lung adenocarcinoma), OA (ovarian cancer), RIRC (kidney renal clear cell carcinoma), UCEC (uterine corpus endometrial carcinoma), STAD (Stomach adenocarcinoma), LIHC (liver hepatocellular carcinoma), etc. was also higher than corresponding control groups (Figures 1(d) and 1(e)). In addition, with the help of “Pathological Stage Plot” module of GEPIA2, we observed increased expression of CKS1B in most tumors with disease progression, especially in ACC (adrenocortical carcinoma), KICH, and KIPR (kidney renal papillary cell carcinoma) (Figure 1(f), Figure S1E).

### 3.2. High Expression of CKS1B Correlates with Tumor Prognosis

**Figure 2:**
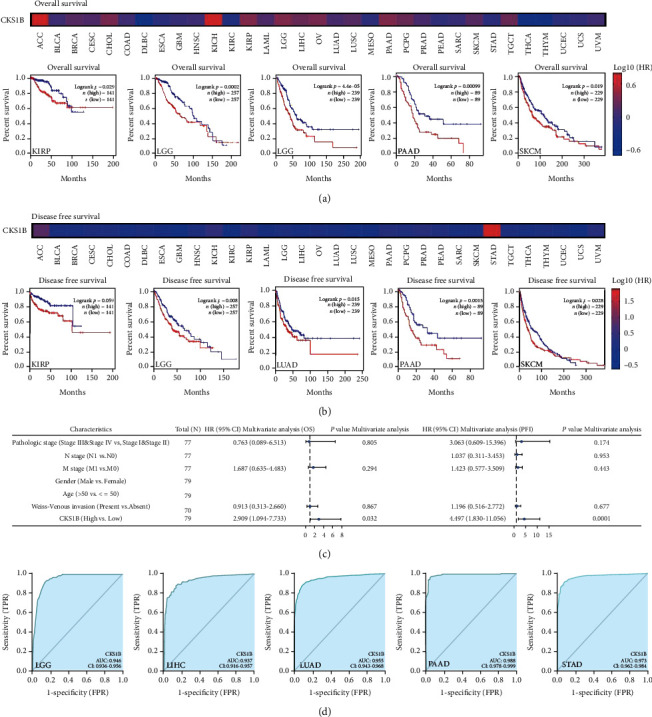
Relationship between CKS1B and survival prognosis. (a) Overall survival and (b) disease-free survival of different tumors based on CKS1B expression level (GEPIA2). (c) Forest plot of multivariate Cox regression analysis of ACC patients. (d) Predictive value of CKS1B expression for diagnosis in LGG, LIHC, LUAD, PAAD, and STAD patients.

**Figure 3:**
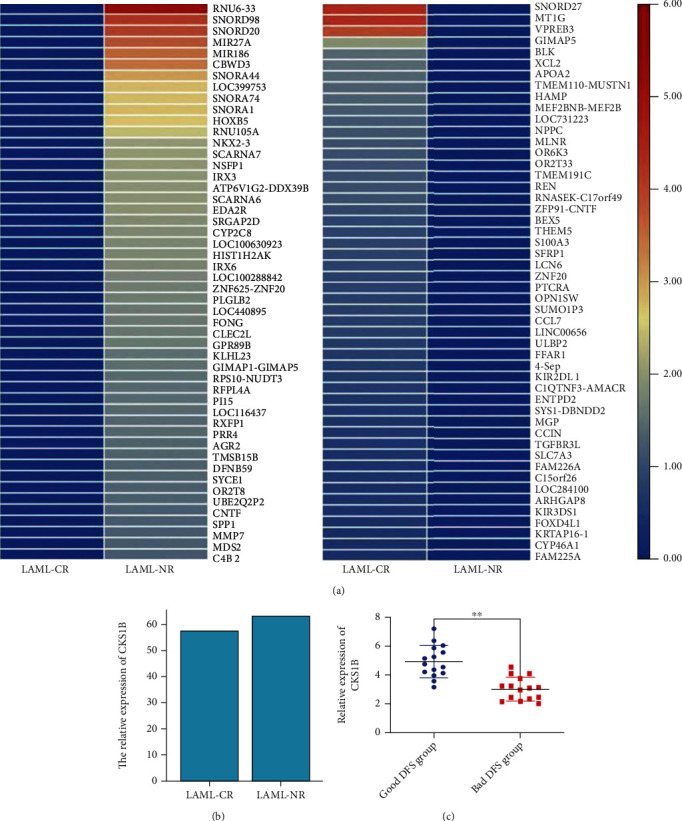
Expression levels of CKS1B in LAML and GEM tissue specimens. (a) RNA sequencing results in LAML showed CKS1B was not among the top 50 differentially expressed genes in the remission (CR) and nonremission (NR) groups after chemotherapy. Although specific data indicated CKS1B was higher in the NR group than that of the CR group (63.5 vs. 57.42), the results showed no statistical difference. (b) RT-qPCR results in GEM showed CKS1B mRNA in patients with good DFS was higher than that in patients with bad DFS.

Tumor cases were divided into high CKS1B expression group and low CKS1B expression group. The correlation between CKS1B and prognosis of patients with different tumors was studied by GEPIA2. As shown in Figures 2(a) and 2(b), highly expressed CKS1B was linked to poor OS and DFS in KIRP, LGG (brain lower grade glioma), LUAD, PAAD (pancreatic adenocarcinoma), and SKCM (skin cutaneous melanoma) (all *p* < 0.05). Interestingly, it was not associated with OS in LAML and LUSC. Moreover, high CKS1B expression was even meant better OS in KIRC (kidney renal clear cell carcinoma) (*p* = 0.026) and better DFS in GBM (glioblastoma multiforme) (*p* = 0.046) (Figure S2A and S2B). To verify this conclusion, on one hand, we collected bone marrow samples from LAML patients and divided them into remission (CR) group and nonremission (NR) group according to the degree of bone marrow remission after chemotherapy. By RNA sequencing, CKS1B was not found among the top 50 differentially expressed genes between the two groups. More specific data showed that although CKS1B in NR group was higher than that in CR group (63.5 vs. 57.42), the difference was not statistically significant (*p* = 0.2083) (Figure 3(a)). On the other hand, through retrospective analysis of clinical data, GEM patients were divided into good prognosis and bad prognosis groups according to DFS, and 30 samples were selected (15 cases in each group). RT-qPCR results showed that CKS1B mRNA in patients with good DFS was higher than that in patients with bad DFS (*p* = 0.0006) (Figure 3(b)). These data indicated that the prognostic significance of CKS1B expression level in different tumor types was not completely the same. Besides, we specifically discussed the predictive value of CKS1B for clinical outcomes in subgroups of ACC, and results were shown in Figure 2(c): high expression of CKS1B was an independent risk factor for OS (HR = 2.909, *p* = 0.032) and progression-free interval (PFI) (HR = 4.497, *p* = 0.001).

In order to evaluate the clinical diagnostic value of CKS1B, we also calculated the area under ROC curve of LGG, LIHC (liver hepatocellular carcinoma), LUAD, PAAD, STAD, BRCA, COAD, ESCA (esophageal carcinoma), LUSC (lung squamous cell carcinoma), OV (ovarian serous cystadenocarcinoma), READ (rectum adenocarcinoma), KIRC, and GBM, most of which were above 0.9, indicating that CKS1B has high sensitivity and specificity for the diagnosis of these tumors (Figure 2(d) and Figure S2C).

### 3.3. CKS1B Correlates with Tumor Immune Infiltration

**Figure 4:**
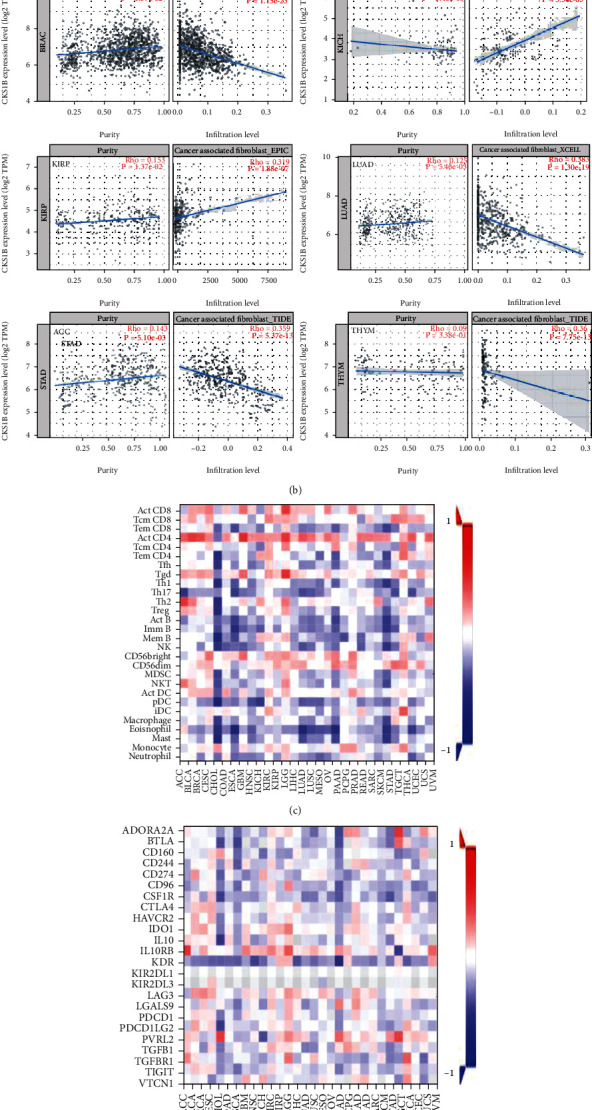
Relationship between CKS1B and tumor immune infiltration. ((a) and (b)) Correlation between CKS1B expression and cancer-associated fibroblasts infiltration levels based on different algorithms. (c) The heat map of the relationship between CKS1B and TILs in different tumors (red means positive correlation, and blue means negative correlation). ((d) and (e)) The heat maps of correlation between CKS1B and immunosuppressive factors and immunostimulatory factors. (f) CKS1B was positively associated with infiltrating levels of Act_CD4 in GEM and BLCA, Tgd in LGG, Th2 in SKCM, but negatively related to Th17 infiltrating in ACC and UCEC.

Immune system plays a crucial role in the occurrence, development, and treatment of tumors [17]. Tumor-infiltrating immune cells are believed to be able to independently predict tumor metastasis and prognosis [18–20]. Considering the upregulation of CKS1B was associated with a variety of tumor progression and prognosis, we speculated CKS1B might be involved in tumor immune response. To confirm this hypothesis, we did a series of comparisons by TIMER and GEPIA2 databases and observed a statistically positive correlation between CKS1B expression and CAFs infiltration in ACC, KICH, and KIRP, but a negative correlation in BRCA, LUAD, LUSC, STAD, and THYM (thymoma) (Figure 4(a)). The scatter plots based on one of XCELL, MCPCOUNTER, TIDE, and EPIC algorithms were shown in Figure 4(b). Moreover, we explored the role of CKS1B in immune regulation by ESTIMATE database. The heat maps about CKS1B and tumor-infiltrating lymphocytes (TILs), immunosuppressive factors, and immunostimulatory factors were presented in Figures 4(c)–4(e), respectively.  Figure 4(f) was another scatter plot reflecting CKS1B and certain TILs in specific tumors. For example, CKS1B was negatively correlated with Th17 infiltration in ACC (*r* = −0.455, *p* = 3.1*e* − 05) and UCEC (*r* = −0.401, *p* = 2.2*e* − 16), but positively correlated with Act_CD4 infiltration in GEM (*r* = 0.504, *p* = 3.1*e* − 05) and BLCA (*r* = 0.462, *p* = 2.2*e* − 16). Besides, heat maps of CKS1B expression with B lymphocytes, T lymphocytes, chemokines, and chemokine receptors were shown in Figure S3A-D.

### 3.4. Enrichment Analysis of CKS1B-Related Partners

**Figure 5:**
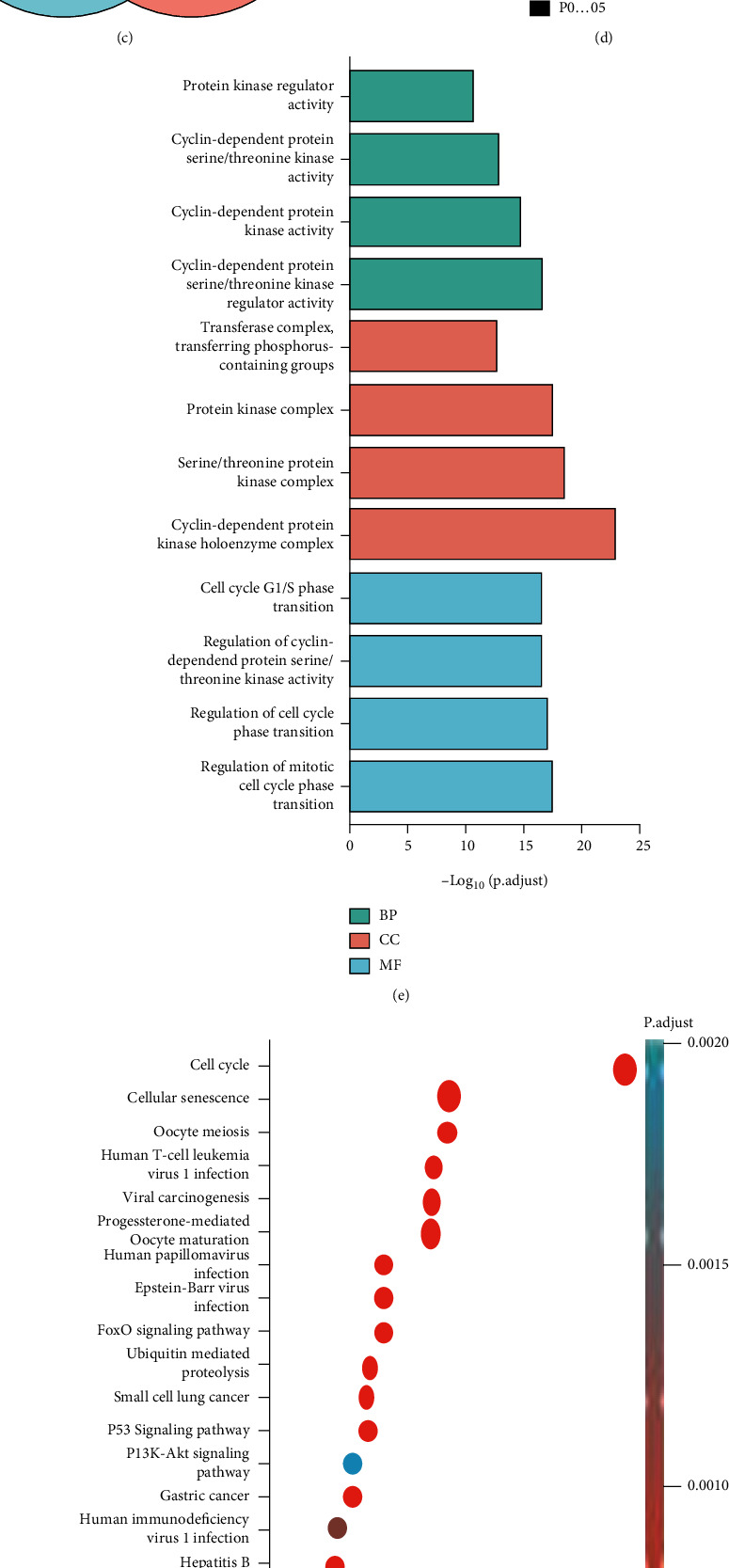
CKS1B-related gene enrichment analysis. (a) The binding protein map targeting CKS1B based on STRING tool. (b) Protein interaction network based on GeneMANIA database. (c) Venn diagram of the cross-analysis of above two results. (d) The expression of 10 screened common genes in various tumors. (e) GO enrichment and (f) KEGG enrichment analysis of CKS1B-related differentially expressed genes (DEGs). (g) Correlation analysis between CKS1B expression and screened common genes, including CDK1, CCNA2, CCNB1, CCNB2, CKS2, and SKP2. (h) Functional annotation of CKS1B-associated DEGs in ACC.

To further investigate the mechanism of CKS1B in tumorigenesis, we attempted to screen out the binding protein map targeting CKS1B by STRING tool (Figure 5(a)) and draw a protein interaction network by GeneMANIA database (Figure 5(b)). A cross-analysis of the above two sets of data revealed that there were 10 common members, namely, CCN2, CCNB1, CCNB2, CDK1, CDK2, CDK3, CDKN1A, CKS2, SKP1, and SKP2 (Figure 5(c)). The expression of these genes in different tumors was presented as a heat map (Figure 5(d)). As shown in Figure 5(g), CKS1B was positively associated with CDK1 (*r* = 0.67), CCN2 (*r* = 0.64), CCNB1 (*r* = 0.68), CCNB2 (*r* = 0.69), CKS2 (*r* = 0.64), and CKP2 (*r* = 0.48) (all *p* < 0.001). Moreover, the GO data in Figure 5(e) demonstrated that “cell cycle regulation” and “protein kinase activity regulation” were involved in the influence of CKS1B on tumor pathogenesis. KEGG data in Figure 5(f) indicated that most of these selected genes were linked to cell cycle, cell senescence, and viral infection (such as Epstein-Barr virus, HPV virus, and hepatitis B virus). FOXO, P53, and PI3K-Akt were the main participating molecules and signaling pathway.

To specifically evaluate the function of CKS1B-related differentially expressed genes (DEGs), we used GSEA for enrichment analysis. As shown in Table 1 and Figure 5(h), CKS1B-related DEGs were mainly enriched in drug metabolism related clusters, such as cytochrome p450 (NES = −2.414, *p*.adj = 0.018, and FDR = 0.012) (Figure 5(h) a) and glucuronidation (NES = −2.225, *p*.adj = 0.018, and FDR = 0.012) (Figure 5(h) b); cell proliferation-related clusters (Figure 5(h) c), such as G2-M checkpoint (NES = 2.807, *p*.adj = 0.018, and FDR = 0.012) (Figure 5(h) d) and mitotic spindle checkpoints (NES = 2.850, *p*.adj = 0.018, and FDR = 0.012) (Figure 5(h) e); and apoptosis related clusters, such as PD-1 signal pathway (NES = −2.222, *p*.adj = 0.018, and FDR = 0.012) (Figure 5(h) f).

### 3.5. Genetic Alteration Analysis of CKS1B

**Figure 6:**
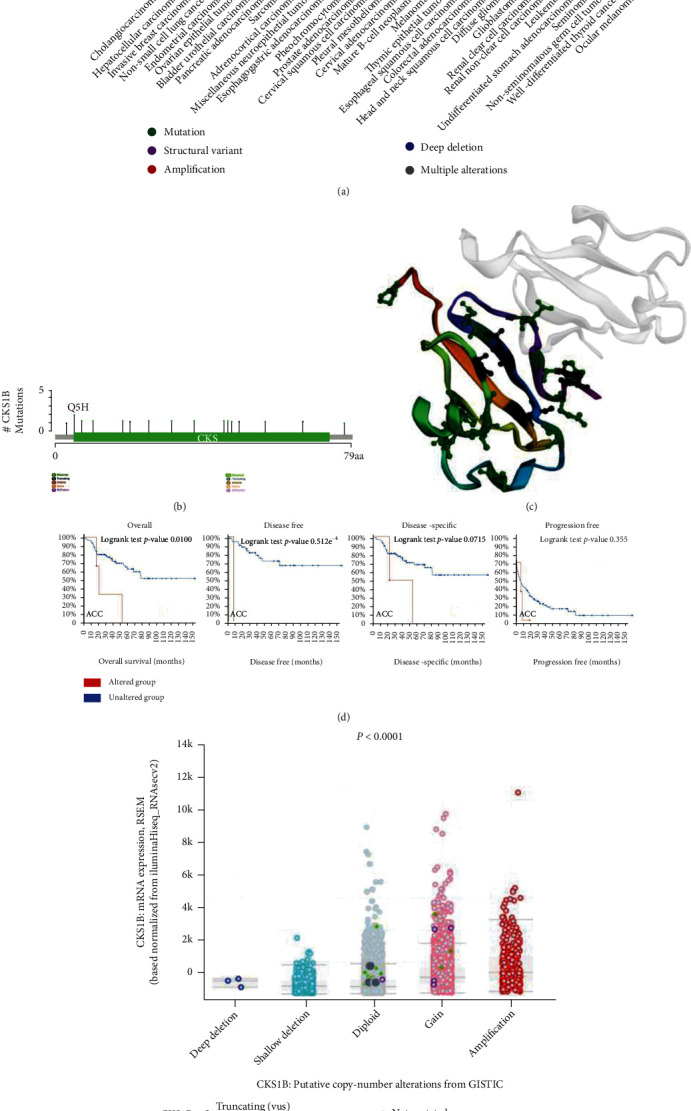
Mutation feature and prognosis significance of CKS1B in tumors. (a) The alteration frequency with mutation type, (b) mutation site, and (c) 3D structure of CKS1B. (d) The potential correlation between CKS1B mutation status and overall survival, disease-specific survival, disease-free, and progression-free survival of ACC based on cBioPortal tool. (e) The association between CKS1B copy number and mRNA expression.

The total frequency of CKS1B genetic alteration in patients with 33 tumor types was 3.54% (388/10953), and the top five tumors with the highest frequency were CHOL (cholangiocarcinoma) (16.67%), LIHC (11.56%), BRCA (9.5%), nonsmall cell lung cancer (9.19%), and UCEC (8.77%). On the contrary, CKS1B genetic variation was hardly observed in KIRC, leukemia, undifferentiated STAD, seminoma, nonseminomatous germ cell tumors, well-differentiated thyroid carcinoma, and ocular melanoma. “Amplification” was the most common type of genetic variation in all tumor cases. In addition, “mutation” in CHOL, COAD, HNSC (head and neck squamous carcinoma), and “structural variant” in pleural mesothelioma also had a high incidence (Figure 6(a)). The location, type, case number of CKS1B genetic variation, and 3D structure of CKS1B protein were further presented in Figures 6(b) and 6(c), respectively. We then investigated the potential association between CKS1B alteration and survival outcomes in tumor patients. Take ACC for instance, patients with altered CKS1B showed a worse OS (*p* = 0.016) and DFS (*p* = 9.542*E* − 4), but not the disease-specific (*p* = 0.0715) and progression-free survival (*p* = 0.355), compared with patients without CKS1B alteration (Figure 6(d)). The dot plot in Figure 6(e) indicated the relationship between the copy number of CKS1B and mRNA expression. It could be seen that the mRNA expression level of ACC samples with CKS1B deletion was lower than that of CKS1B amplification.

### 3.6. CKS1B DNA Methylation Analysis Data

**Figure 7:**
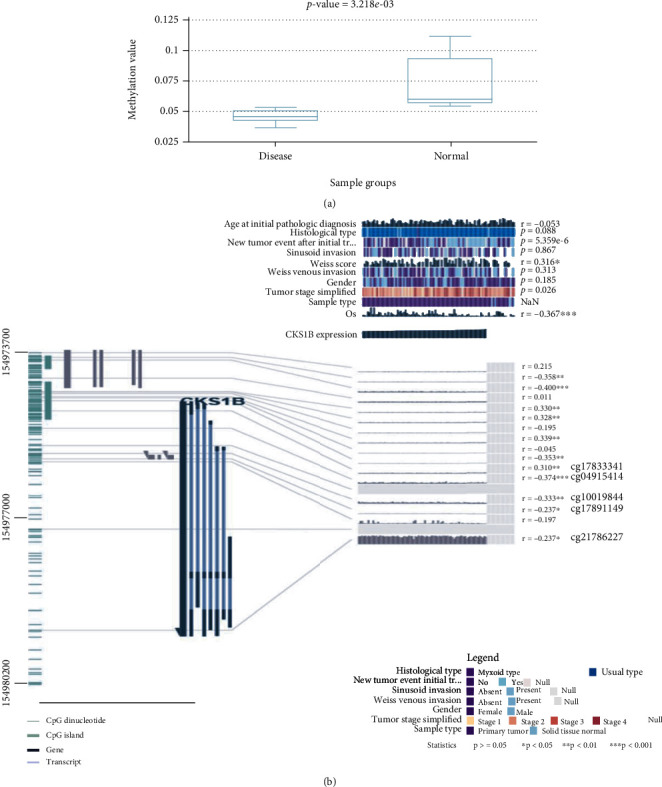
CKS1B DNA methylation analysis. (a) Differences of CKS1B methylation in ACC and corresponding normal tissues based on DiseaseMeth version 2.0. (b) The methylation sites of CKS1B DNA sequence were associated with gene expression based on MEXPRESS. CKS1B expression was illustrated by the blue line in the center of the plot. Pearson's correlation coefficients and *p* values for methylation sites and query gene expression were shown on the right side.

Methylation analysis result in ACC demonstrated that CKS1B methylation was significantly lower in tumor than corresponding normal tissues (Figure 7(a)). Beyond that, we also found 4 methylation sites (cg04915414, cg10019844, cg17891149, and cg21786227) which negatively correlated with CKS1B expression and 1 methylation site (cg17833341) which positively correlated with CKS1B expression in DNA sequence (Figure 7(b)).

### 3.7. CKS1B Correlates with Tumor Mutational Burden and Microsatellite Instability

**Figure 8:**
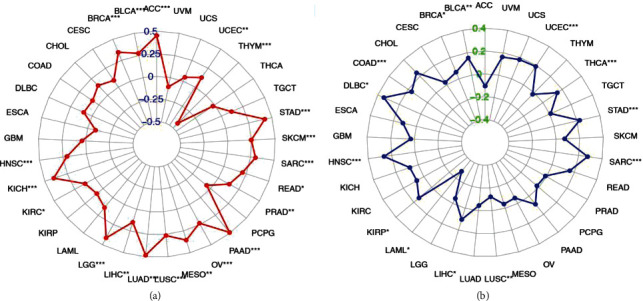
Correlation between CKS1B and TMB/MSI in different tumors. (a) Correlation between TMB and CKS1B expression. (b) Correlation between MSI and CKS1B expression. ^∗^*p* < 0.05, ^∗∗^*p* < 0.01, and ^∗∗∗^*p* < 0.001.

Tumor mutation burden (TMB) is the total number of mutations per million bases in the coding region of gene exons that encode specific tumor cell proteins, including insertions, substitutions, deletions, and other forms of mutations [21]. It is also an emerging biomarker for the prediction of immunotherapy in certain tumors, such as lung cancer, malignant melanoma, and bladder cancer [22–24]. Microsatellite instability (MSI) is a genetic change. In the process of normal cell proliferation, there is a complete DNA mismatch repair system, which can detect the replication errors of microsatellite sequence in time and quickly correct it, so that the microsatellite sequence can be replicated in high fidelity, thus, maintaining the stability of it [25]. Due to the DNA mismatch repair defects in process of tumorigenesis, errors in the replication cannot be detected in time, causing insertion or deletion of repeated units, or changes in the length of microsatellite sequences, which eventually leads to MSI [26]. A large number of clinical observations, retrospective studies, and meta-analysis have confirmed that MSI is closely related to tumor prognosis [27]. Here, we analyzed the relationship between CKS1B expression and TMB/MSI in the TCGA database. As shown in Table 2 and Figure 8(a), CKS1B was negatively correlated with TMB in THYM, but positively correlated with it in ACC, UCEC, STAD, SKCM, SARC (Sarcoma), etc. (all *p* < 0.05). Besides, CKS1B was also negatively correlated with MSI in LUSC and LAML, but positively correlated with it in UCEC, THCA, STAD, SARC, LIHC, KIRP, HNSC, DLBC (lymphoid neoplasm diffuse large B-cell lymphoma), COAD, BRCA, and BLCA (bladder urothelial carcinoma) (Table 3 and Figure 8(b), all *p* < 0.05). In combination with the foregoing, our results indicated that CKS1B had both tumor prognosis and therapeutic effect prediction value. This point deserves further study.

## 4. Discussion

CKS1B, also known as cell cycle-dependent protease regulatory subunit, is a small molecule protein (9KD) encoded by CKS1 gene in the lq21 region of human chromosome and participate a lot of important physiological and pathological processes. Recently, more and more scholars have discovered that CKS1B is closely related to the occurrence and development of malignant tumors. For example, Fujita et al. found CKS1B protein was highly expressed in nonsmall cell lung cancer patients [12]. Shrestha et al. confirmed both CKS1B mRNA and protein in gastric cancer cells were significantly higher than those in normal control cells [11]. Liu et al. reported CKS1B in breast cancer was associated with patient's age, estrogen, and progesterone receptor levels and increased with malignant degree [15]. Besides, CKS1B was also found to be upregulated in patients with prostate cancer, colorectal cancer, leukemia, retinoblastoma, and other malignant diseases or animal models [10, 28, 29]. Therefore, CKS1B is generally regarded as a cancer-promoting factor. However, most studies of CKS1B have focused on a single disease, and pan-cancer analysis of it from a holistic perspective has not been reported yet. Here, we searched several of the most important databases, such as TCGA, TIMER, and GEPIA, to comprehensively summarize CKS1B gene expression, genetic changes, methylation modifications, and prognosis analysis in different tumors.

Our results revealed that although CKS1B was highly expressed in most tumors, its survival and prognostic significance varied among them. For example, high CKS1B expression was associated with poor OS and DFS in KIRP, LGG, LUAD, PAAD, and SKCM. In view of this, identifying high-risk patients as soon as possible, formulating personalized treatment plans, and strengthening regular follow-up of these patients are expected to improve their prognosis. However, CKS1B showed no correlation with OS of LUSC and LAML. More even, its high expression was related to favorable OS in RIRC and better DFS in GEM. Our RNA sequencing in LAML and RT-qPCR in GEM also confirmed this. While whether the current evidence based on databases could fully and truly reflect the prognostic significance of CKS1B in other tumors need to be further verified by more basic experiments.

We also investigated the relationship between CKS1B and TMB and MSI. It has been demonstrated that these two indicators can predict patient's response to multiple drugs, especially immune checkpoint inhibitors [30–32]. In this work, CKS1B was shown to be positively associated with TMB and MSI in UCEC, STAD, LIHC, etc., so we speculated that these types of tumors may benefit from immune therapy. CKS1B may be used as an evaluation index of chemotherapeutic responsiveness and provide reference value for clinical drug guidance of some tumors. In addition, we compared the difference of DNA methylation status in the nonpromoter region of CKS1B. In cases of ACC, we found CKS1B methylation was significantly lower in tumor tissues than adjacent normal tissues. The potential role of CKS1B DNA methylation in tumourgenesis is worthy of further study.

Occurrence and progression of tumors are not only caused by genetic changes of tumor cells themselves but also the microenvironment also plays a key role in this process [33, 34]. Tumor microenvironment includes cells and extracellular matrix, among which CAF is one of the most important members and accounting for about 50% of total number of cells [35]. CAFs can produce a variety of cytokines and metabolites through direct contact or paracrine and involve in tumor proliferation, metastasis, angiogenesis, drug resistance, etc. [36–38]. Here, we found CKS1B was positively correlated with CAFs infiltration in ACC, KICH, and KIRP, but negatively in BRCA, LUAD, STAD, and THYM. Previous studies have reported that high expression of CKS1B could induce drug resistance of lung cancer cells to cisplatin and adriamycin, but it is unclear whether CAFs are involved [14, 39]. Although we are temporarily unable to provide more specific data on CKS1B and CAFs in the LUAD research, we believe that the results of this paper can provide a new idea for future research on CKS1B and lung cancer drug resistance to a certain extent. The mechanism by which CKS1B and CAFs affect tumor microenvironment will be an interesting research direction. Moreover, we analyzed the association between CKS1B and expression of TILs, immunosuppressive factors, and immunostimulatory factors in tumor microenvironment. For example, in LGG, CKS1B was positively correlated with Tgd, IL10RB, CD276, and CD48. This was consistent with the conclusion reported by Zou et al. that CD48 was highly expressed and had a poor prognosis in the malignant progression of glioma [40]. Our study provides useful information about the involvement of CKS1B in immune regulation.

## 5. Conclusions

Our first pan-cancer analysis of CKS1B demonstrated a statistical association between CKS1B and tumor clinical prognosis, immune cell infiltration, DNA methylation, tumor mutation burden, and microsatellite instability across multiple tumors. It is helpful to understand the role of CKS1B from a holistic perspective. However, there are some limitations of our studies. In the future, we will focus on verifying these obtained data through basic experiments to better understand the mechanism and regulatory network of CKS1B.

## Figures and Tables

**Table 1:** Gene set enrichment analysis of CKS1B.

Gene set name	NES	*p* value	FDR *q*-val
KEGG_DRUG_METABOLISM_CYTOCHROME_P450	-2.414	0.002	0.012
KEGG_RETINOL_METABOLISM	-2.368	0.002	0.012
KEGG_METABOLISM_OF_XENOBIOTICS_BY_CYTOCHROME_P450	-2.317	0.002	0.012
KEGG_STEROID_HORMONE_BIOSYNTHESIS	-2.289	0.002	0.012
KEGG_ASTHMA	-2.267	0.002	0.012
KEGG_ASCORBATE_AND_ALDARATE_METABOLISM	-2.252	0.002	0.012
REACTOME_GLUCURONIDATION	-2.225	0.002	0.012
REACTOME_PD_1_SIGNALING	-2.222	0.002	0.012
KEGG_ALLOGRAFT_REJECTION	-2.209	0.002	0.012
WP_PREGNANE_X_RECEPTOR_PATHWAY	-2.198	0.002	0.012
REACTOME_G2_M_CHECKPOINTS	2.807	0.003	0.012
REACTOME_MITOTIC_G1_PHASE_AND_G1_S_TRANSITION	2.819	0.003	0.012
REACTOME_MITOTIC_SPINDLE_CHECKPOINT	2.85	0.003	0.012
REACTOME_RESOLUTION_OF_SISTER_CHROMATID_COHESION	2.857	0.003	0.012
REACTOME_M_PHASE	2.901	0.003	0.012
REACTOME_MITOTIC_PROMETAPHASE	2.914	0.003	0.012
WP_CELL_CYCLE	2.951	0.003	0.012
KEGG_CELL_CYCLE	2.976	0.003	0.012
WP_RETINOBLASTOMA_GENE_IN_CANCER	2.979	0.003	0.012
REACTOME_CELL_CYCLE_CHECKPOINTS	3.11	0.003	0.012

**Table 2:** Correlation analysis regarding the association of CKS1B expression and TMB.

Cancer type	Cor	*p* value	Sig
ACC	0.451	<0.001	∗∗∗
BLCA	0.274	<0.001	∗∗∗
BRCA	0.344	<0.001	∗∗∗
CESC	0.089	0.134	
CHOL	0.146	0.395	
COAD	0.082	0.105	
DLBC	0.097	0.568	
ESCA	-0.087	0.277	
GBM	0.041	0.622	
HNSC	0.207	<0.001	∗∗∗
KICH	0.399	0.001	∗∗∗
KIRC	0.123	0.025	∗
KIRP	0.086	0.155	
LAML	0.141	0.272	
LGG	0.415	<0.001	∗∗∗
LIHC	0.149	0.005	∗∗
LUAD	0.483	<0.001	∗∗∗
LUSC	0.264	<0.001	∗∗∗
MESO	0.355	0.001	∗∗
OV	0.233	<0.001	∗∗∗
PAAD	0.492	<0.001	∗∗∗
PCPG	-0.054	0.472	
PRAD	0.133	0.003	∗∗
READ	0.207	0.017	∗
SARC	0.301	<0.001	∗∗∗
SKCM	0.248	<0.001	∗∗∗
STAD	0.422	<0.001	∗∗∗
TGCT	0.118	0.159	
THCA	-0.025	0.582	
THYM	-0.433	<0.001	∗∗∗
UCEC	0.126	0.004	∗∗
UCS	0.047	0.729	
UVM	-0.105	0.355	

**Table 3:** Correlation analysis regarding the association of CKS1B expression and MSI.

Cancer type	Cor	*p* value	Sig
ACC	-0.107	0.349	
BLCA	0.153	0.002	∗∗
BRCA	0.063	0.045	∗
CESC	0.025	0.669	
CHOL	0.242	0.155	
COAD	0.164	0.001	∗∗∗
DLBC	0.344	0.017	∗
ESCA	0.117	0.142	
GBM	0.031	0.704	
HNSC	0.266	<0.001	∗∗∗
KICH	0.075	0.553	
KIRC	0.068	0.212	
KIRP	0.142	0.017	∗
LAML	-0.278	0.022	∗
LGG	-0.049	0.270	
LIHC	0.103	0.047	∗
LUAD	-0.045	0.312	
LUSC	-0.123	0.006	∗∗
MESO	-0.038	0.736	
OV	-0.053	0.384	
PAAD	0.089	0.244	
PCPG	-0.021	0.779	
PRAD	-0.011	0.807	
READ	0.158	0.052	
SARC	0.277	<0.001	∗∗∗
SKCM	0.073	0.113	
STAD	0.223	<0.001	∗∗∗
TGCT	0.009	0.913	
THCA	0.15	0.001	∗∗∗
THYM	-0.018	0.842	
UCEC	0.191	<0.001	∗∗∗
UCS	0.181	0.182	
UVM	0.162	0.150	

## Data Availability

Some of the original data can be obtained directly from TGGA, OCOMINE, and other databases, further inquiries (RNA sequencing and PCR data) can be directed to the corresponding author.

## Supplementary Materials

Supplementary Materials Figure S1: CKS1B expression in different types of human tumors. The basal expression level of CKS1B in different (a) blood cells, (b) tumor cell lines, and (c) tumor tissues using Consensus database. (d) The expression of CKS1B in paired tumors and normal tissues of CHOL, ESCA, KIRP, READ, COADREAD, THCA, KICH, and PRAD. (e) Correlations between CKS1B and tumor stages in BRCA, LIHC, and THCA patients based on GEPIA2. ^∗^*p* < 0.05; ^∗∗^*p* < 0.01; ^∗∗∗^*p* < 0.001. Figure S2: correlation of CKS1B expression level with survival prognosis. (a) Overall survival and (b) disease-free survival of different tumors based on CKS1B expression level (GEPIA2). (c) Predictive value of CKS1B expression for diagnosis in BRCA, COAD, ESCA, LUSC, OV, READ, KIRC, and GEM patients. Figure S3: correlation of CKS1B expression with tumor immune infiltration. Heat maps of the relationship between CKS1B expression and (a) B lymphocytes, (b) T lymphocytes, (c) chemokines, and (d) chemokine receptors.
