# Supplementary material for: A Pan-Cancer Analysis of Clinical Prognosis and Immune Infiltration of CKS1B in Human Tumors
Source: Biomed Res Int. 2021 Nov 20;2021:5862941. doi: 10.1155/2021/5862941 (PMC8627364; doi:10.1155/2021/5862941)
Supplement: Supplementary Materials — Figure S1: CKS1B expression in different types of human tumors. The basal expression level of CKS1B in different (a) blood cells, (b) tumor cell lines, and (c) tumor tissues using Consensus database. (d) The expression of CKS1B in paired tumors and normal tissues of CHOL, ESCA, KIRP, READ, COADREAD, THCA, KICH, and PRAD. (e) Correlations between CKS1B and tumor stages in BRCA, LIHC, and THCA patients based on GEPIA2. ∗p < 0.05; ∗∗p < 0.01; ∗∗∗p < 0.001. Figure S2: correlation of CKS1B expression level with survival prognosis. (a) Overall survival and (b) disease-free survival of different tumors based on CKS1B expression level (GEPIA2). (c) Predictive value of CKS1B expression for diagnosis in BRCA, COAD, ESCA, LUSC, OV, READ, KIRC, and GEM patients. Figure S3: correlation of CKS1B expression with tumor immune infiltration. Heat maps of the relationship between CKS1B expression and (a) B lymphocytes, (b) T lymphocytes, (c) chemokines, and (d) chemokine receptors. [file 5862941.f1.pdf]

**Supplementary Material**

**Figure S1** CKS1B expression in different types of human tumors.

The basal expression level of CKS1B in different (A) blood cells, (B) tumor cell lines, and (C) tumor tissues using Consensus database. (D) The expression of CKS1B in paired tumors and normal tissues of CHOL, ESCA, KIRP, READ, COADREAD,THCA, KICH and PRAD. (E) Correlations between CKS1B and tumor stages in BRCA, LIHC and THCA patients based on GEPIA2. * $P < 0.05$; ** $P < 0.01$; *** $P < 0.001$.

**Figure S2** Correlation of CKS1B expression level with survival prognosis.

(A) Overall survival and (B) disease-free survival of different tumors based on CKS1B expression level (GEPIA2). (C) Predictive value of CKS1B expression for diagnosis in BRCA, COAD, ESCA, LUSC, OV, READ, KIRC and GEM patients.

**Figure S3** Correlation of CKS1B expression with tumor immune infiltration.

Heat maps of the relationship between CKS1B expression and (A) B lymphocytes, (B) T lymphocytes, (C) chemokines and (D) chemokine receptors.

Figure S1

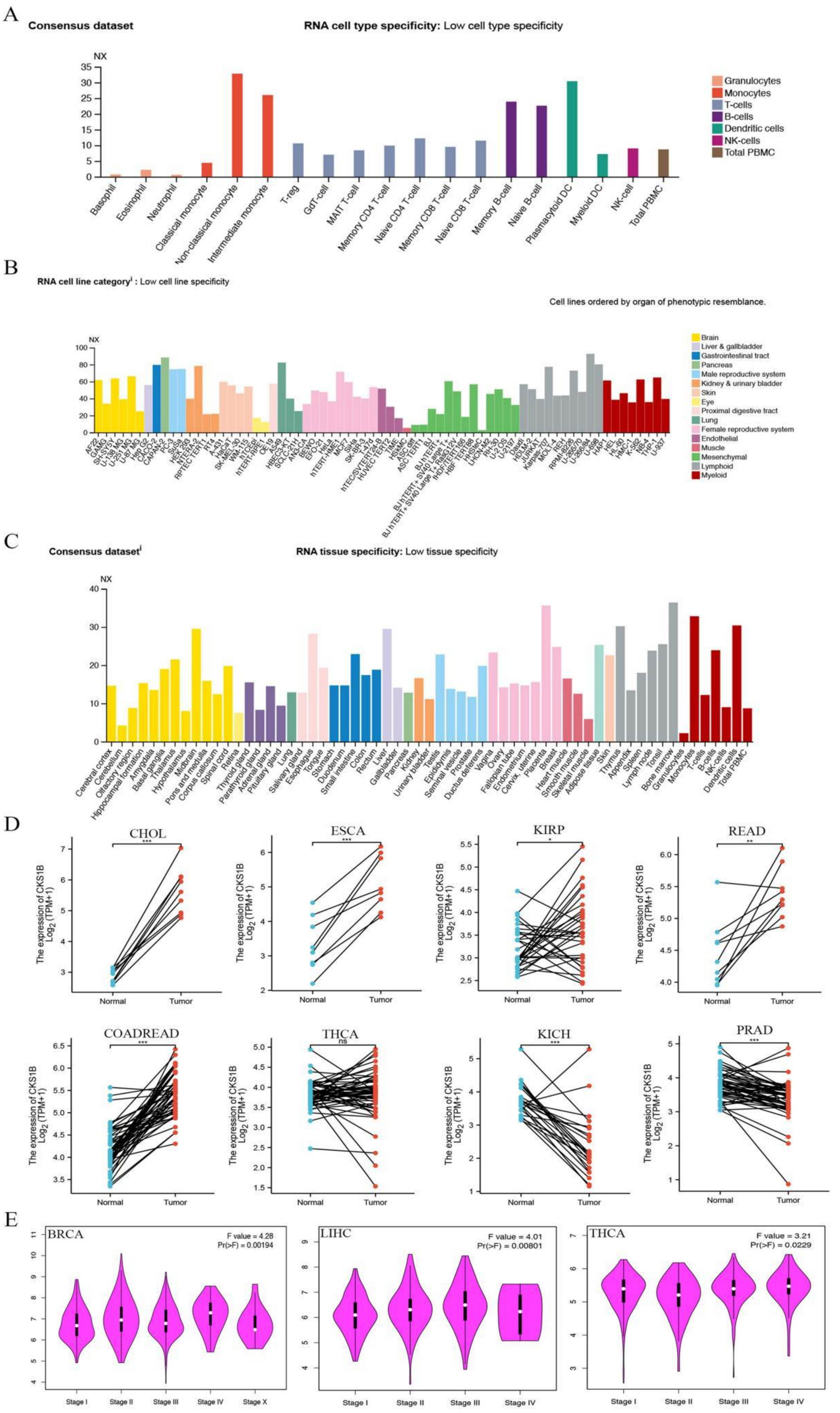
A
Consensus dataset
RNA cell type specificity: Low cell type specificity
NX
Granulocytes
Monocytes
T-cells
B-cells
Dendritic cells
NK-cells
Total PBMC
Basophil
Eosinophil
Neutrophil
Classical monocyte
Non-classical monocyte
Intermediate monocyte
T-reg
GdT-cell
MAIT T-cell
Memory CD4 T-cell
Naive CD4 T-cell
Memory CD8 T-cell
Naive CD8 T-cell
Memory B-cell
Naive B-cell
Plasmacytoid DC
Myeloid DC
NK-cell
Total PBMC
B
RNA cell line category : Low cell line specificity
Cell lines ordered by organ of phenotypic resemblance.
NX
Brain
Liver & gallbladder
Gastrointestinal tract
Pancreas
Male reproductive system
Kidney & urinary bladder
Skin
Eye
Proximal digestive tract
Lung
Female reproductive system
Endothelial
Muscle
Mesenchymal
Lymphoid
Myeloid
C
Consensus dataset
RNA tissue specificity: Low tissue specificity
NX
Cerebral cortex
Cerebellum
Olfactory region
Hippocampal formation
Amygdala
Basal ganglia
Thalamus
Hypothalamus
Midbrain
Pons and medulla
Corpus callosum
Spinal cord
Retina
Thyroid gland
Parathyroid gland
Adrenal gland
Pituitary gland
Lung
Salivary gland
Esophagus
Tongue
Stomach
Duodenum
Small intestine
Colon
Rectum
Liver
Gallbladder
Pancreas
Kidney
Urinary bladder
Testis
Epididymis
Seminal vesicle
Prostate
Ductus deferens
Vagina
Ovary
Fallopian tube
Endometrium
Cervix, uterine
Placenta
Breast
Heart muscle
Smooth muscle
Skeletal muscle
Adipose tissue
Skin
Thymus
Appendix
Spleen
Lymph node
Tonsil
Bone marrow
Granulocytes
Monocytes
T-cells
B-cells
NK-cells
Dendritic cells
Total PBMC
D
CHOL
ESCA
KIRP
READ
COADREAD
THCA
KICH
PRAD
The expression of CKS1B Log2 (TPM+1)
Normal
Tumor
***
*
**
ns
E
BRCA
F value = 4.28
Pr(>F) = 0.00194
Stage I
Stage II
Stage III
Stage IV
Stage X
LIHC
F value = 4.01
Pr(>F) = 0.00801
THCA
F value = 3.21
Pr(>F) = 0.0229

Figure S2

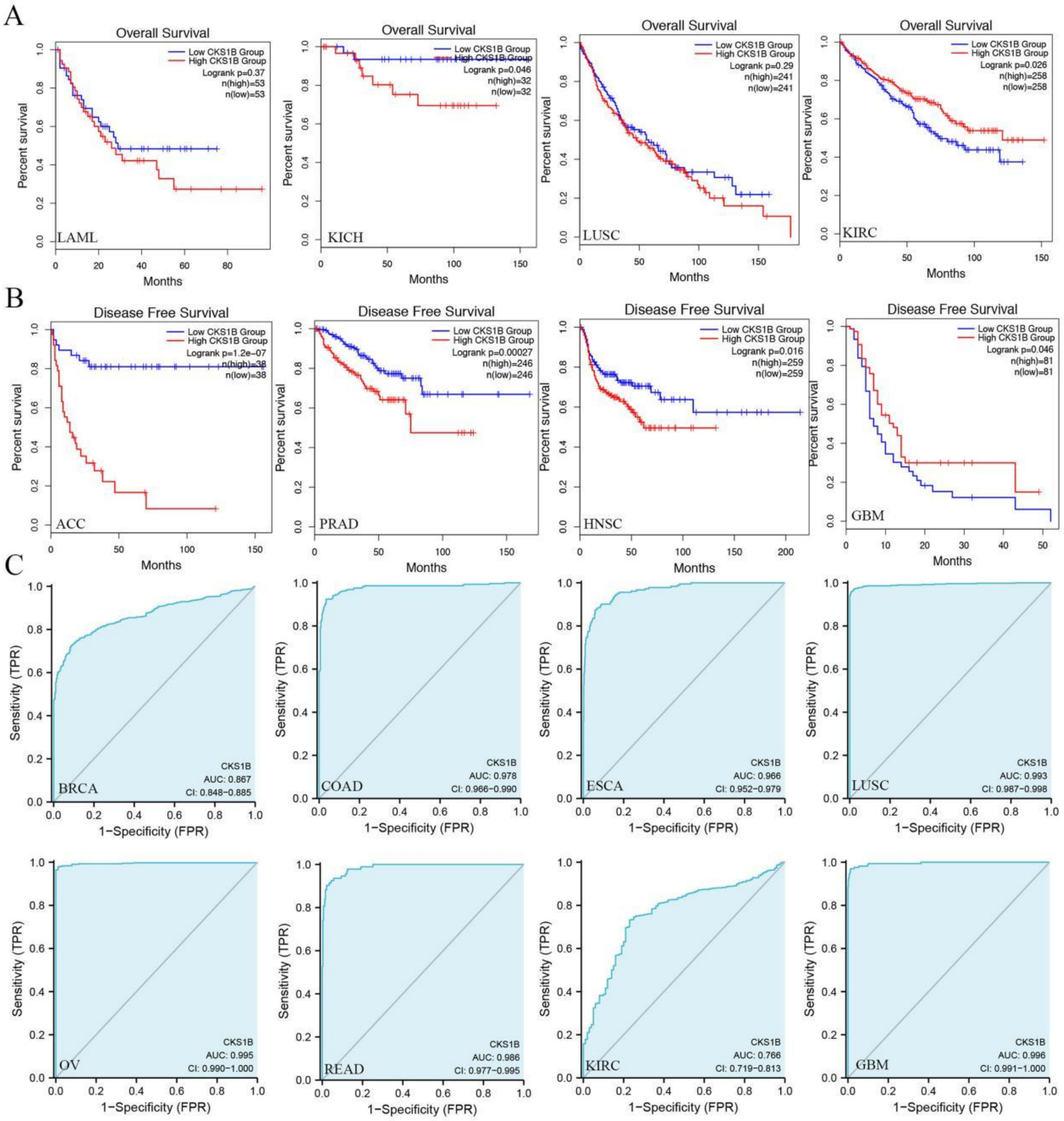

A
Overall Survival
Low CKS1B Group
High CKS1B Group
Logrank p=0.37
n(high)=53
n(low)=53
LAML
Logrank p=0.046
n(high)=32
n(low)=32
KICH
Logrank p=0.29
n(high)=241
n(low)=241
LUSC
Logrank p=0.026
n(high)=258
n(low)=258
KIRC
Percent survival
Months
B
Disease Free Survival
Logrank p=1.2e−07
n(high)=38
n(low)=38
ACC
Logrank p=0.00027
n(high)=246
n(low)=246
PRAD
Logrank p=0.016
n(high)=259
n(low)=259
HNSC
Logrank p=0.046
n(high)=81
n(low)=81
GBM
C
Sensitivity (TPR)
1−Specificity (FPR)
CKS1B
BRCA
AUC: 0.867
CI: 0.848−0.885
COAD
AUC: 0.978
CI: 0.966−0.990
ESCA
AUC: 0.966
CI: 0.952−0.979
LUSC
AUC: 0.993
CI: 0.987−0.998
OV
AUC: 0.995
CI: 0.990−1.000
READ
AUC: 0.986
CI: 0.977−0.995
KIRC
AUC: 0.766
CI: 0.719−0.813
GBM
AUC: 0.996
CI: 0.991−1.000

Figure S3

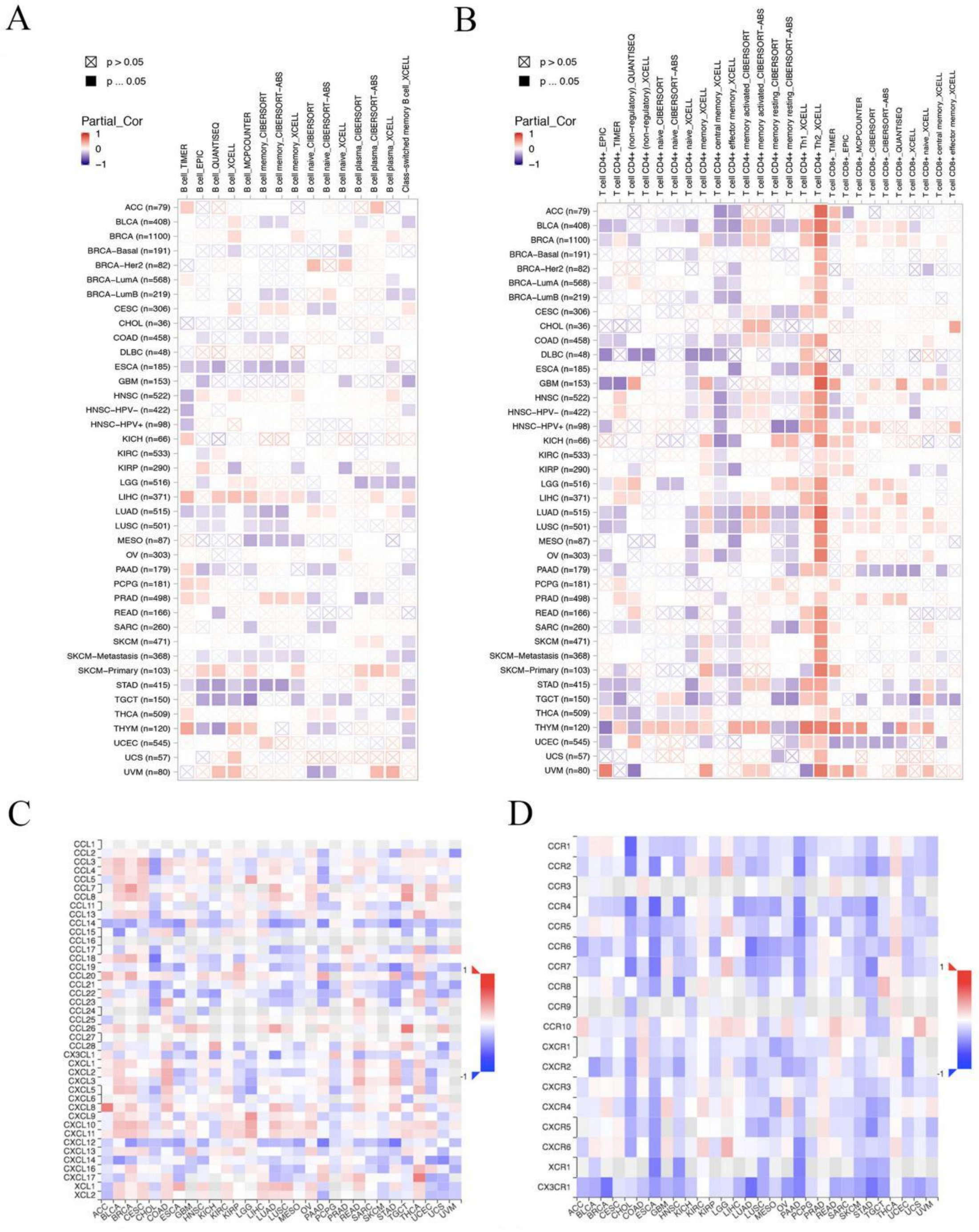
A
p > 0.05
p ... 0.05
Partial_Cor
1
0
−1
B cell_TIMER
B cell_EPIC
B cell_QUANTISEQ
B cell_XCELL
B cell_MCPCOUNTER
B cell memory_CIBERSORT
B cell memory_CIBERSORT−ABS
B cell memory_XCELL
B cell naive_CIBERSORT
B cell naive_CIBERSORT−ABS
B cell naive_XCELL
B cell plasma_CIBERSORT
B cell plasma_CIBERSORT−ABS
B cell plasma_XCELL
Class−switched memory B cell_XCELL
ACC (n=79)
BLCA (n=408)
BRCA (n=1100)
BRCA−Basal (n=191)
BRCA−Her2 (n=82)
BRCA−LumA (n=568)
BRCA−LumB (n=219)
CESC (n=306)
CHOL (n=36)
COAD (n=458)
DLBC (n=48)
ESCA (n=185)
GBM (n=153)
HNSC (n=522)
HNSC−HPV− (n=422)
HNSC−HPV+ (n=98)
KICH (n=66)
KIRC (n=533)
KIRP (n=290)
LGG (n=516)
LIHC (n=371)
LUAD (n=515)
LUSC (n=501)
MESO (n=87)
OV (n=303)
PAAD (n=179)
PCPG (n=181)
PRAD (n=498)
READ (n=166)
SARC (n=260)
SKCM (n=471)
SKCM−Metastasis (n=368)
SKCM−Primary (n=103)
STAD (n=415)
TGCT (n=150)
THCA (n=509)
THYM (n=120)
UCEC (n=545)
UCS (n=57)
UVM (n=80)
B
p > 0.05
p ... 0.05
Partial_Cor
1
0
−1
T cell CD4+_EPIC
T cell CD4+_TIMER
T cell CD4+ (non−regulatory)_QUANTISEQ
T cell CD4+ (non−regulatory)_XCELL
T cell CD4+ naive_CIBERSORT
T cell CD4+ naive_CIBERSORT−ABS
T cell CD4+ naive_XCELL
T cell CD4+ memory_XCELL
T cell CD4+ central memory_XCELL
T cell CD4+ effector memory_XCELL
T cell CD4+ memory activated_CIBERSORT
T cell CD4+ memory activated_CIBERSORT−ABS
T cell CD4+ memory resting_CIBERSORT
T cell CD4+ memory resting_CIBERSORT−ABS
T cell CD4+ Th1_XCELL
T cell CD4+ Th2_XCELL
T cell CD8+_TIMER
T cell CD8+_EPIC
T cell CD8+_MCPCOUNTER
T cell CD8+_CIBERSORT
T cell CD8+_CIBERSORT−ABS
T cell CD8+_QUANTISEQ
T cell CD8+_XCELL
T cell CD8+ naive_XCELL
T cell CD8+ central memory_XCELL
T cell CD8+ effector memory_XCELL
ACC (n=79)
BLCA (n=408)
BRCA (n=1100)
BRCA−Basal (n=191)
BRCA−Her2 (n=82)
BRCA−LumA (n=568)
BRCA−LumB (n=219)
CESC (n=306)
CHOL (n=36)
COAD (n=458)
DLBC (n=48)
ESCA (n=185)
GBM (n=153)
HNSC (n=522)
HNSC−HPV− (n=422)
HNSC−HPV+ (n=98)
KICH (n=66)
KIRC (n=533)
KIRP (n=290)
LGG (n=516)
LIHC (n=371)
LUAD (n=515)
LUSC (n=501)
MESO (n=87)
OV (n=303)
PAAD (n=179)
PCPG (n=181)
PRAD (n=498)
READ (n=166)
SARC (n=260)
SKCM (n=471)
SKCM−Metastasis (n=368)
SKCM−Primary (n=103)
STAD (n=415)
TGCT (n=150)
THCA (n=509)
THYM (n=120)
UCEC (n=545)
UCS (n=57)
UVM (n=80)
C
CCL1
CCL2
CCL3
CCL4
CCL5
CCL7
CCL8
CCL11
CCL13
CCL14
CCL15
CCL16
CCL17
CCL18
CCL19
CCL20
CCL21
CCL22
CCL23
CCL24
CCL25
CCL26
CCL27
CCL28
CX3CL1
CXCL1
CXCL2
CXCL3
CXCL5
CXCL6
CXCL8
CXCL9
CXCL10
CXCL11
CXCL12
CXCL13
CXCL14
CXCL16
CXCL17
XCL1
XCL2
ACC BLCA BRCA CESC CHOL COAD ESCA GBM HNSC KICH KIRC KIRP LGG LIHC LUAD LUSC MESO OV PAAD PCPG PRAD READ SARC SKCM STAD TGCT THCA UCEC UCS UVM
1
-1
D
CCR1
CCR2
CCR3
CCR4
CCR5
CCR6
CCR7
CCR8
CCR9
CCR10
CXCR1
CXCR2
CXCR3
CXCR4
CXCR5
CXCR6
XCR1
CX3CR1
ACC BLCA BRCA CESC CHOL COAD ESCA GBM HNSC KICH KIRC KIRP LGG LIHC LUAD LUSC MESO OV PAAD PCPG PRAD READ SARC SKCM STAD TGCT THCA UCEC UCS UVM
1
-1
